# Does Excess Weight Interfere with Bone Mass Accumulation during Adolescence?

**DOI:** 10.3390/nu5062047

**Published:** 2013-06-06

**Authors:** Luciana Nunes Mosca, Valéria Nóbrega da Silva, Tamara Beres Lederer Goldberg

**Affiliations:** Postgraduate Program in Gynecology, Obstetrics, and Mastology, Discipline of Adolescent Medicine, Department of Pediatrics, Botucatu School of Medicine, UNESP, São Paulo State University, Botucatu, SP 18618-970, Brazil; E-Mails: lunutricionista@yahoo.com.br (L.N.M.); valerianutry@gmail.com (V.N.S.)

**Keywords:** adolescents, bone mineral density, obesity, osteoporosis, overweight, risk factors

## Abstract

Obesity and osteoporosis are important global health problems characterized by increasing prevalence with high impact on morbidity and mortality. The objective of this review was to determine whether excess weight during adolescence interferes with bone mass accumulation. If bone mineral gain can be optimized during puberty, adults are less likely to suffer from the devastating complications of osteoporosis. The increased fracture risk in obese children has also been attributed to a lower bone mass for weight compared to non-obese children. Thus, adiposity present in this age group may not result in the protection of bone mass, in contrast to what has been observed in adults. However, studies involving adolescents have reported both protective and detrimental effects of obesity on bone. The results and mechanisms of these interactions are controversial and have not been fully elucidated, a fact highlighting the extreme relevance of this topic and the need to monitor intervening and interactive variables.

## 1. Introduction

Obesity is a chronic disease associated with a range of comorbidities, which has been a major focus of researchers, health agencies and departments, and populations around the world. Among different concepts, obesity can be defined as excessive general or local accumulation of body fat or adipose tissue that exerts adverse effects on the health of affected individuals. Overweight is defined when body weight exceeds the ideal weight in relation to height [1,2].

The presence of obesity in adolescence is a risk factor for obesity in adult life [3,4]. In addition, the diagnosis of obesity is believed to have various repercussions on bone metabolism during adolescence and may be a possible determinant of bone mass accumulation and bone fragility [5,6]. Since more than 90% of adult bone mass is acquired during childhood and adolescence [7], impairment of bone accumulation and peak bone mass during this period can have long-term repercussions on bone health in adults and older adults [8].

Excess weight is one of the most serious public health problems and its prevalence in childhood and adolescence has increased dramatically over the past few decades in both developed and developing countries [9]. Data published by the World Health Organization (WHO) show that at least 1 billion people have excess weight and 300 million of them are obese [10]. A recent study conducted in the United States [11] using data from the National Health and Nutrition Examination Surveys (NHANES) found that, between 2009 and 2010, 69.2% of adults were overweight (BMI ≥ 25 kg/m^2^) and 35.9% were obese (BMI ≥ 30 kg/m^2^). Projections based on national surveys conducted over the last decades estimate that, in 2025, 40% of the population in the United States, 30% of the population in England, and 20% of the population in Brazil will be obese [12]. The latest data show that 24.9% of American children and adolescents are overweight. Although the prevalence of obesity is relatively high (16.9%), it has been stable in both genders when comparing data obtained in 2007–2008 with the results of the 2009–2010 NHANES. However, an alarming situation is observed for boys aged 12 to 19 years whose BMI is above the 97th percentile of 12.3%, a condition called extremely obese [13]. In European countries such as Spain, Italy and Greece [13,14,15], the prevalence of overweight and obesity ranges from 20% to 30% among children and adolescents. A similar situation has been observed in non-Western countries. A recent meta-analysis of studies conducted over the past 10 years in India reported a prevalence of excess weight and obesity higher than 12% and 3%, respectively [16]. In Brazil, the prevalence of overweight/obesity among boys and girls aged 10 to 19 years increased from 3.9% and 7.6%, respectively, in 1974/1975 to 21.7% and 19% in 2008/2009 [17].

A number of studies have investigated the possible triggers of obesity in adolescents [4,14,18,19]. The results revealed an important genetic contribution based on the analysis of genotypes in obese children and adolescents. According to Genome-Wide Association Studies (GWAS), 28 loci showed consistent effects associated with the altered phenotype, whereas 32 loci were found to be related to obesity in adults [20]. Other factors may also play a role. In this respect, environmental factors and lifestyle changes such as inadequate eating habits, increased fast food consumption and low levels of habitual physical activity (>4 h computer use and watching television) exert a strong influence on weight gain, compromising functions of the organism and nutritional status [4,14,18,19].

The consequences of excess weight on adolescent health are a matter of concern since obesity-associated metabolic alterations which, until recently, were more common among adults, are now frequently seen in the adolescent population. These alterations include dyslipidemia, arterial hypertension and glucose intolerance, which are risk factors for type 2 diabetes mellitus, cardiovascular diseases, and orthopedic problems (e.g., osteoarthritis and gout) [3,21,22,23,24].

Several factors influence bone mass gain, including gender, race, hereditary factors , body weight, diet, habitual physical activity, and hormones involved in bone calcification. Body weight, which basically comprises fat and lean mass, has been identified as one of the main determinants of bone mineral content (BMC) and can exert diverse influences in the same skeletal region. Body weight gain interferes with both the acquisition and loss of bone mass and is directly associated with the risk of overweight or obesity [25,26].

Since obesity and osteoporosis are serious public health problems throughout the world and obesity is seen at increasingly younger ages, as well as the fact that the higher life expectancy of the population has led to an increase in the prevalence of osteoporosis and resulting fractures, the interaction of these processes requires strategies for the diagnosis, prevention, control, and treatment of these conditions. Within this context, the objective of the present study was to perform a narrative and detailed literature review of the possible effects of excess body fat on bone mass in adolescents. 

## 2. Methods

For the purpose of the study, the MEDLINE and PubMed databases and the Scielo and Lilacs databases were searched for articles published in English and Portuguese, respectively, over a period of 10 years (January 2002 to October 2012) using the following MeSH terms: obesity, overweight, osteopenia/osteoporosis, and adolescence.

## 3. Adolescence: Anthropometry and Evaluation of Bone Mineral Content

Adolescence is a period in life characterized by intense physiological, psychosocial, behavioral, cultural and emotional transformations that occur concomitantly or sequentially. This phase comprises important physical growth and development, including maturation of the body when the child turns into an adult, a period recognized as puberty [27]. This process is characterized by visible physiological changes such as gain in body weight and height, fat and muscle mass gain, variations in bone mass, and the development of secondary sexual characteristics [28,29,30]. The WHO defines persons ranging in age from 10 to 19 years as adolescents [31].

A fundamental process that occurs during puberty is bone mineral acquisition. Bone mineral content increases gradually during childhood and then accelerates during adolescence in both genders, *i.e.*, the rate of bone formation exceeds the rate of bone resorption, resulting in bone modeling and remodeling [32,33,34,35]. After the cessation of growth, bone mass continues to increase for some years until reaching a peak, which can be defined as the maximum amount of bone mass a person accumulates from birth to skeletal maturity [36,37,38]. In a study involving healthy white Canadian children and adolescents aged 8 to 14 years (113 boys and 115 girls), Bailey *et al.* [36] selected those who would still reach peak height velocity and peak BMC velocity (*n* = 113) for longitudinal analysis by annual lumbar spine, proximal femur and whole-body dual-energy X-ray absorptiometry (DXA). Mean age of peak bone mineral accretion analyzed by whole-body DXA was 14.0 (SD = 1.0) years for boys and 12.5 (SD = 0.9) years for girls, whereas peak height velocity occurred at 13.4 (SD = 1.0) years in boys and at 11.8 (SD = 0.9) in girls. These findings confirm that maximum bone mass gain continues even after peak height velocity has been reached [36]. Boot *et al.* [38] studied 501 healthy subjects ranging in age from 13 to 29 years, including 360 girls and 141 boys, using longitudinal and cross-sectional data of this population. In that study, lumbar and whole-body peak bone mass occurred between 18 and 20 years in girls and between 20 and 23 years in boys.

Determination of BMI is recommended for the diagnosis of overweight and obesity. The BMI for age should be compared to reference curves such as those published by the Centers for Disease Control and Prevention (CDC) [39] and the WHO [40]. According to the CDC, adolescents with a BMI above the 85th and 95th percentiles are considered to be at risk of overweight and obesity, respectively. The WHO defines adolescents with a BMI above the 97th percentile as obese [39,40].

Bone mineral density (BMD) is a noninvasive measure used for the evaluation of bone health and can be classified according to the technique used or bone site studied. This measure has been applied to establish normal patterns that differ between different ethnic groups and that permit the precise and accurate quantification of bone mass according to age group, gender and biological maturity status [41]. BMD (g/cm^2^) and BMC (g) can be measured with precision by DXA in the lumbar spine and proximal femur. Using the appropriate software, many DXA devices equally measure BMD in the forearm and whole body, providing precise data of whole-body composition [41].

Since DXA calculates areal, and not volumetric, BMD and since the bone area does not increase at the same proportion as bone volume during growth, the true BMD of children and adolescents might be overestimated for large bones and underestimated for small bones, considering that the organism of children and adolescents suffers major somatic transformations and bones therefore vary widely in size [42,43]. Therefore, some authors prefer the use of BMC for growing individuals [6].

Recently, peripheral quantitative computed tomography (pQCT) was proposed as an imaging tool for the study of both trabecular and cortical bones in adolescents and adults. This proposal is based on the capacity of this method to evaluate volumetric bone density and bone geometry, providing a better understanding of bone strength [44,45]. In a study on adolescent girls using pQCT, Farr *et al.* [45] observed that the higher the fat content of calf and thigh muscle fibers, the lower the volumetric BMD and, consequently, bone strength. Fat content evaluated at these sites may therefore represent a risk factor for the presence of bone fragility and fractures during the phase of growth. Although the contrast resolution of pQCT is lower than that of magnetic resonance imaging, the low costs of this method favor its use for the evaluation of large population samples. However, studies are needed to determine reference ranges and measurement sites and to standardize devices and softwares. Since children and adolescents are in the phase of growth, the exact and most consistent site of measurement needs to be determined [44]. Variable precision of the results depending on the site and limb used for measurement has been reported in adults [46].

## 4. Nutrients and Bone Mass

Dietary calcium intake has been the focus of studies evaluating the role of nutrients in the determination of peak bone mass [47,48,49]. In addition to this nutrient, adequate supply of phosphorus, magnesium, energy, protein, zinc, copper, and vitamin C is necessary to prevent classical deficiencies. These nutrients are obtained from food or supplemental sources. Furthermore, sun exposure as well as adequate dietary and supplemental intake are needed to maintain adequate vitamin D levels [50]. The recognition of the effect of nutritional factors on bone mineralization permits early intervention in order to prevent the occurrence of osteopenia and osteoporosis. Although these diseases manifest in older adults, they can have their origin in childhood and adolescence [37,47].

The recommended intake of calcium is based on the relationship between calcium intake and maximal calcium retention during growth. The Dietary Reference Intake (DRI) [51] for calcium is 1300 mg/day for adolescent boys and girls. There are certain impediments that prevent adolescents from achieving excellent bone health. Most adolescents do not consume the recommended daily amounts of calcium and the main cause of inadequate calcium intake is an overall decline in the consumption of dairy products during these years [52,53]. Many adolescents no longer drink milk for various reasons, some are intolerant and others do not like its taste or consider milk to be a “child beverage”, and in most cases milk is replaced with other beverages such as juice and soft drinks [54].

In a recent study, Hill *et al.* [49] provided a mathematical model that suggested calcium intake by adolescents to be the most important external factor for skeletal calcium retention, which accounted for 15% and 21.7% of the variation in calcium retention in adolescent girls and boys, respectively [49]. If this intake is lower than the DRI [51], impaired calcium retention would lead to suboptimal BMC, increasing the risk of fractures in adolescents at the time of peak bone mass. The inflection point for calcium intake determined in that study was 1640.2 mg/day. Skeletal calcium retention increased with BMI up to this point. Above this plateau, calcium retention increased with BMI, but not with increasing calcium intake. Obese adolescents ingesting about 800 mg calcium/day, the mean intake observed among American adolescents and a value lower than the DRI, retained only 3.4 mg/day more calcium than normal weight adolescents, which may be considered of low magnitude. The authors therefore hypothesized that obese adolescents would be at a lower risk of fractures if they consume the DRI for calcium, a question that can be answered only by longitudinal studies. Also according to this model for obese adolescents, the inclusion of variables other than BMI and adequate calcium intake, such as weight, height, fat percentage, Tanner stage, parathormone (PTH), and 25-hydroxyvitamin D [25(OH)D], was unable to explain gains in skeletal calcium retention. Further investigation is needed to confirm or refute the hypothesis set forth by the model of Hill *et al.* [49].

Vitamin D is a liposoluble pro-hormone and its precursors, vitamin D3 (cholecalciferol) and vitamin D2 (ergocalciferol), have been extensively studied, but not in terms of the effects on health outcomes in children and adolescents. Vitamin D precursors are converted into 25(OH)D (calcidiol), which corresponds to the main circulating fraction and is an indicator of vitamin D status [55]. The concentrations of 25(OH)D have been shown to be reduced in obese subjects, even adolescents. In contrast to non-obese subjects, this vitamin can be sequestered and stored in subcutaneous fat, reducing its release into the bloodstream [55,56,57]. No consensus exists regarding the cut-off points used to define vitamin D deficiency, but levels <20 ng/mL (50 nmol/L) are considered to indicate deficiency or insufficiency. In view of these considerations and since a supply of 400 IU vitamin D/day may not be sufficient to increase blood concentrations to levels >40 nmol/L, the recommended daily vitamin D intake for adolescents is 600 IU/day [51].

Lenders *et al.* [58], studying 58 obese adolescents seen at Boston Children’s Hospital, evaluated factors associated with vitamin D deficiency and bone mass and observed that 29% of the adolescents were deficient in 25(OH)D. Nevertheless, PTH, BMC and BMD were within the normal range for age and gender. In addition, a large number of the adolescents with 25(OH)D deficiency presented higher fat mass and fat mass percentage, as well as high leptin levels. Lower osteocalcin concentrations were observed in these adolescents when compared to non-deficient subjects (*p* < 0.05). The authors emphasized that their study was the first involving obese adolescents with vitamin D deficiency in the absence of elevated PTH and with normal bone mass, and suggested the need for further investigations to evaluate the relationship between factors regulating energy and bone metabolism in obese adolescents [58].

## 5. Effects of Adiposity on Bone Mass

Various mechanisms have been proposed to explain the complex relationship between adipose tissue and bone. The physiopathological role of adipose tissue in bone homeostasis is probably related to the participation of some adipokines in bone remodeling. These molecules are released from fat cells and some of them interfere with both bone formation and resorption. Since bone cells express specific hormone receptors, bone tissue has been suggested to be an endocrine organ [59,60]. Adipose tissues also secrete inflammatory cytokines such as interleukin 6 (IL-6) and tumor necrosis factor alpha (TNF-α) [61]. The altered production of these proinflammatory markers can have adverse metabolic effects and cardiovascular repercussions. IL-6 and TNF-α also promote bone resorption by stimulating the differentiation of osteoclasts [62]. All of these molecules, including resistin, adiponectin, leptin and IL-6, affect energy homeostasis in humans and might be involved differently in bone metabolism, thus contributing to the complex relationship between adipose tissue and bone tissue [62].

The relationship between adipose tissue and bone probably results in a homeostatic feedback system in which adipokines and molecules secreted by osteoblasts and osteoclasts are part of an active bone-adipose axis. However, the mechanisms involved in these events remain unclear [63]. In the case of postmenopausal women, leptin has been shown to be positively correlated with increased BMD at all sites analyzed. In contrast, in adult premenopausal women, leptin only showed a positive and strong correlation with lumbar spine BMD, whereas the correlation with femoral and whole-body BMD was weak. The same was observed for men. However, in a systematic review conducted by Biver *et al.* [64], leptin was no longer correlated with BMD after adjustment for weight, BMI and fat mass in many studies. The results reported so far are highly divergent and multiple regression models rarely identified leptin as an independent predictor of BMD, explaining no more than 1% to 7% of the variability in BMD.

With respect to adiponectin, studies involving adults, men and postmenopausal women have demonstrated a negative correlation between this adipokine and BMD, which was also observed after adjustment for the same factors as used in the studies on leptin (weight, BMI, and fat mass). In obese adolescents followed up for weight loss over a period of one year, adiponectin exerted an effect on bone formation through osteoblast activation and inhibition of osteoclasts, with the observation of bone mass gain evaluated based on BMC. However, the increase of BMC observed in that study indicates a reduction of insulin resistance as a result of weight loss in obese adolescents, since insulin resistance compromises bone mass gain [65]. In a large study conducted in England on adolescents with a mean age of 9.9 years submitted to DXA and evaluated again by pQCT at 15.5 years, who were part of the Avon Longitudinal Study of Parents and Children (ALSPAC), Sayers *et al.* [66] also demonstrated an inverse association between adiponectin and BMC gain, bone area and BMD, which was stronger in boys than in girls. These findings suggest puberty to be an attenuating factor of the effect of adiponectin on bone development in girls. However, the authors emphasized that total adiponectins were measured and that isoforms with different functions may exert different effects on bone mass [66]. On the other hand, none of the studies evaluating resistin and ghrelin found evidence of a correlation between these hormones and BMD in adult women or men [62,64]. Taken together, these results indicate a very weak correlation of adipokines and ghrelin with BMD, probably because of the interference of specific parameters of body composition such as fat mass [65]. There is no convincing evidence supporting correlations of resistin, visfatin or ghrelin with BMD, although the number of studies involving adults is small. In view of these findings, Biver *et al.* [64] concluded that adipokines affect bone metabolism differently.

Particularly in adolescents, the effects of obesity on bone mass have not been fully established. This knowledge is important since impaired acquisition of peak bone mass may increase the risk of low BMD and fractures due to bone fragility in adult life [67]. Roemmich *et al.* [68] studied the relationship between leptin, a hormone produced and secreted by adipose tissue, and bone mineralization in children and adolescents by evaluating the association between adiposity and bone mass. Serum leptin concentration increased with increasing fat accumulation. However, the authors concluded that, in boys and girls, serum leptin concentrations were not related to BMC irrespective of chronological age. Further studies are needed to confirm the possible relationship between adiposity and adequate bone mineralization at all ages. More recently, Prado *et al.* [69] evaluated the relationship of body composition, leptin, glucose levels, insulinemia and insulin resistance (evaluated by Homeostasis Model Assessment and the Quantitative Insulin Sensitivity Check Index), with BMC and BMD in a group of 109 Brazilian obese adolescents. The results demonstrated a negative association of insulinemia, leptin levels and markers of insulin resistance with BMD, suggesting that the parameters analyzed play a significant role in bone metabolism.

## 6. Relation between Obesity and Bone Mass

The effects of obesity on bone mass have not been fully elucidated. According to Migliaccio *et al.* [63], if obesity is defined in premenopausal and postmenopausal women on the basis of BMI, it protects against bone loss and fractures. However, if obesity is diagnosed based on body fat percentage, it may be a risk factor for osteopenia/osteoporosis. The authors concluded that different degrees of obesity may interfere differently with bone mass.

A number of studies have evaluated the effect of obesity on bone mass using DXA, obtaining measures in relation to age, bone size, and body size [26,70]. Some of the results indicate the presence of normal bone or increased BMC in obese children [71,72], whereas other studies reported a reduction of bone size and bone mass in these children [73,74].

Obesity that develops during childhood and adolescence may accelerate skeletal maturation [6]. Leonard *et al.* [70] investigated BMC by DXA in 132 obese (BMI ≥ 95th percentile) and non-obese (BMI < 85th percentile) subjects from Philadelphia according to Tanner stage. The authors observed that obesity was associated with greater height-for-age, advanced bone maturation, and a significant increase of the parameters analyzed in obese subjects compared to normal weight subjects. These associations continued to be significant after adjustment for degree of pubertal development and gender. These results suggest that obesity may exert a protective effect on bone mass [70]. In view of the controversies, Young *et al.* [75] studied a sample of 285 female twins aged 8 to 25 years and found that lean mass was the main determinant of bone mass acquisition during the early years of puberty and that fat mass assumes a predominant role after the pubertal growth spurt. In a cross-sectional study, Clark *et al.* [76] evaluated the relationship between fat mass and lean mass in children from a large population-based cohort in England. The results showed a positive association between body fat mass and increased bone mass, but this association only became marked by the end of puberty.

Studying the orthopedic complications of excess weight in children and adolescents and the relationship between obesity and osteoporosis, investigators observed that body fat mass has no protective effects on bone mass and that a combination of genetic and environmental factors may exert beneficial effects on obesity (reducing body fat mass) and osteoporosis. Comparison of obese and non-obese adolescents showed greater mobility difficulties, increased anatomical measures, osteomuscular problems and a higher rate of fractures in the obese group, demonstrating the beneficial effects of appropriate body weight on bone health [77,78].

Recent studies have shown that the positive correlation between excess weight and BMD is explained by lean mass and that the relationship between adiposity and BMD might be negative [77,79]. Zhao *et al.* [77] investigated the association between BMD and fat mass in two groups of subjects: 489 Caucasians (45% women) with a mean age of 48 years, and 1988 Chinese subjects (42% menopausal women) with a mean age of 45.1 years. Although BMD was positively correlated with BMI and lean mass, negative correlations were observed between BMD and fat mass in kg and between BMD and percent fat mass, which persisted after adjustment for body weight. Afghani and Goran [26] evaluated 256 Latino children (111 girls and 145 boys), who were particularly vulnerable to excess weight and obesity. The authors observed that, independent of age, weight and Tanner stage, central adiposity was inversely associated with BMC, especially in girls.

Pollock *et al.* [6] investigated the possible influence of fat tissue on bone mass. The authors proposed techniques that reflect the three-dimensional distribution of fat and bone (e.g., computed tomography), since bone densitometry alone is unable to differentiate visceral fat from that located in subcutaneous tissues, or cortical from trabecular bone. In this respect, tomography can provide specific and local answers regarding the effect of excess body fat. Specifically analyzing the evolution of whole-body BMC in adolescents, the authors found no significant differences between adolescents with excess weight who presented no risk factor for metabolic syndrome and those with overweight/obesity who had one or ≥2 risk factors. Next, when controlling for covariates such as age, weight, height and race, BMC was 5.4% lower in adolescents with at least one component of metabolic syndrome when compared to those without any component (*p* = 0.018) and 6.3% when compared to those with ≥2 risk factors (*p* = 0.007), suggesting that excess fat mass, particularly that represented by visceral adiposity, associated with metabolic risk factors had no beneficial effect on bone mass [6]. Janicka *et al.* [80] reported that excess body fat is not beneficial to trabecular bone, predominantly found in vertebrae, or the geometry of cortical bones located in appendicular skeletons.

In an attempt to understand not only the indirect, mechanical effects of fat and lean mass on bone mass, but also the direct nonmechanical association between fat mass and BMD, Lucas *et al.* [81] evaluated weight, height, BMI and fat mass in 868 thirteen-year-old Portuguese girls. Fat mass was measured by bioelectrical impedance analysis and fat area was estimated using a skinfold equation. The authors observed a positive association with BMD, with this effect resulting from the load of body weight on bone mass. However, to evaluate nonmechanical effects, the authors used linear regressions and multivariate models to exclude possible confounding variables that would interfere with the association mediated by fat mass and BMD measured in the nondominant forearm. They found no beneficial effect of increasing adiposity on bone mass [81].

Gracia-Marco *et al.* [79] analyzed the results of a sample of 282 Spanish adolescents from Zaragoza in order to evaluate the relationship between adiposity and bone mass. The authors used the following instruments: DXA for the evaluation of BMC and BMD; air displacement plethysmography for the evaluation of fat mass; analysis of calcium intake; determination of sexual maturation stage; anthropometric assessment (weight, height, BMI), and an uniaxial accelerometer for the evaluation of physical activity. Despite control for confounding factors, the authors observed a higher whole-body BMC in boys with excess weight when compared to non-obese boys, and higher BMC and BMD at most sites analyzed in girls with excess weight when compared to eutrophic girls. The authors suggested that excess fat mass may indirectly increase bone mass in adolescents through an increase of lean mass. However, controlling for lean mass inverted the associations between fat mass and bone mass, which became negative, indicating that fat mass itself has no beneficial effect on bone mass. The process can be explained by two possible mechanisms: obese subjects have larger muscles, a fact resulting in greater bone mass, and the bones of heavier subjects might be more stimulated than those of normal weight subjects. Finally, the authors concluded that cross-sectional studies like their own can only provide suggestive evidence of causal relationships [79].

Finally, since studies in the literature are conflicting, understanding bone mass acquisition in overweight or obese adolescents appears to be an interesting line of research.

## 7. Conclusions

Although the childhood obesity epidemic is an undisputed reality and the commitment of researchers to this topic is beyond doubt, the results reported are controversial and the mechanisms underlying the effect of excess weight on bone mass gain during growth are poorly understood and require further investigation. Maximum bone mass gain occurs during adolescence and adequate eating habits and regular physical activity during this period of life are therefore important to prevent excess weight and its comorbidities, as well as the consequences of excess fat mass on total bone mass acquisition.
